# Pembrolizumab-Induced Hypothyroidism: A Case Report

**DOI:** 10.7759/cureus.41889

**Published:** 2023-07-14

**Authors:** Carlos Gaibor, Rohan Das, Vijayakumari Reddy

**Affiliations:** 1 Internal Medicine, St. Luke's Hospital, Chesterfield, USA; 2 Internal Medicine, Saint Louis University School of Medicine, Saint Louis, USA

**Keywords:** drug-induced hypothyroidism, seronegative myasthenia gravis, myasthenia gravis, hypothyroidism, pembrolizumab

## Abstract

Pembrolizumab is a monoclonal antibody frequently used as immunotherapy for lung cell carcinoma that has been reported to cause hypothyroidism and myasthenia gravis among other, unwanted side effects. Here, we present an interesting case of a 77-year-old male previously diagnosed with lung adenocarcinoma managed with pembrolizumab. Initially, he was admitted after a mechanical fall sustaining a facial laceration and subacute fracture in the nasal bone. However, during the workup, thyroid-stimulating hormone (TSH) was found to be elevated, which was attributed to the history of pembrolizumab usage.

## Introduction

The advent of monoclonal antibody immunotherapies has been a revolutionary development in the field of oncology. Immune checkpoint inhibitors (ICIs) are a prominent class of monoclonal antibodies that have been employed to treat a wide array of malignancies [1]. Programmed death protein 1 (PD-1) is expressed on activated T lymphocytes that recognize programmed death ligands 1 and 2 (PDL-1 and PDL-2), widely expressed by tumor cells in order to evade the immune system [2]. Pembrolizumab (Keytruda), a humanized immunoglobulin G4 (IgG4) monoclonal antibody, is an immune checkpoint inhibitor (ICI) that targets programmed death protein 1 (PD-1), thereby blocking the interaction between PD-1 and PDL-1 and PDL-2, leading to an enhancement of T-cell mediated immune response against tumor cells [3]. Therefore, pembrolizumab and other immunotherapeutic agents have changed the course of metastatic disease, showing promising results, and are currently being used for the treatment of advanced melanoma, non-small cell carcinoma, and solid tumors [4]. Nevertheless, immune checkpoint inhibitors are not void of adverse effects, including autoimmune toxicity caused by the risk of activated T lymphocytes attacking normal tissue generally known as immune-related adverse events (irAEs) that could affect the skin, gastrointestinal tract, liver, and endocrine system among other organ and tissues [5]. Furthermore, ICIs have been linked to a wide spectrum of neurologic immune-related adverse events (irAEs), including meningoencephalitis, myasthenia gravis, and various neuropathies, as well as endocrinology systems such as hypo- or hyperthyroidism [6-10].

## Case presentation

The patient was a 77-year-old male with a past medical history of lung adenocarcinoma managed with pembrolizumab, bilateral deep venous thrombosis (DVT) in the lower extremities currently managed with Eliquis, and seronegative, pembrolizumab-induced myasthenia gravis (MG) currently managed with prednisone and pyridostigmine who presented to the hospital after an acute episode of a mechanical fall after tripping on a rug, causing facial trauma. The patient endorsed having a similar episode of mechanical fall two weeks ago while being hospitalized due to severe anemia that was attributed to his history of malignancy. Initial laboratory tests revealed a hemoglobin of 11.4 g/dL (normal range: 13.6-16.5 g/dL), white blood cell count of 10.7 K/uL (normal range: 4.3-10.0 K/uL), which normalized to 6.4 K/uL the next day, platelets of 177 K/uL (normal range: 140-350 K/uL), sodium of 139 mmol/L (normal range: 137-145 mmol/L), potassium of 3.8 mmol/L (normal range: 3.5-4.9 mmol/L), blood urea nitrogen of 27 mg/dL (normal range: 9-20 mg/dL), and creatinine of 1.29 mg/dL (normal range: 0.70-1.30 mg/dL) (Table 1).

**Table 1:** Laboratory tests

Laboratory	Result	Normal range
White blood cells	10.7 K/uL	4.3-10.0 K/uL
Hemoglobin	11.4 g/dl	13.6-16.5 g/dl
Hematocrit	51.10%	40.0-48.0%
Mean corpuscular volume (MCV)	94.4 fL	82.0-99.0 fL
Mean corpuscular hemoglobin (MCH)	30.2 pg	27.2-32.6 pg
Mean corpuscular hemoglobin concentration (MCHC)	32.0 g/dl	31.5-35.5 g/dl
Red cell distribution width (RDW)	17.20%	11.5-14.5%
Sodium	143 mmol/L	137-145 mmol/L
Potassium	4.4 mmol/L	3.5-4.9 mmol/L
Chloride	107 mmol/L	98-107 mmol/L
Magnesium	1.8 mg/dl	1.6-2.6 mg/dl
Bicarbonate	35 mmol/L	22-30 mmol/L
Blood urea nitrogen (BUN)	36 mg/dl	9-20 mg/dl
Creatinine	1.52 mg/dl	0.7-1.3 mg/dl
Glucose	97 mg/dl	74-106 mg/dl
Calcium	8.9 mg/dl	8.4-10.2 mg/dl
Protein total	6.2 g/dl	6.5-8.6 g/dl
Albumin	3.7 g/dl	3.5-5.0 g/dl
Alkaline phosphatase	86 U/L	38-126 U/L
Bilirubin, total	0.6mg/dl	0.2-1.3 mg/dl
Aspartate aminotransferase (AST)	66 U/L	14-54 U/L
Alanine transaminase (ALT)	53 U/L	≤50 U/L
CK total	135 U/L	55-170 U/L
Thyroid Stimulating Hormone (TSH)	29.8 uUI/ml	0.47-4.68 uUI/ml
Free thyroxine (T4)	0.8 ng/dl	0.8-2.2 ng/dl
Thyroid peroxidase antibody (Ab)	<0.9 IU/ml	0-34 IU/ml

**Figure 1:**
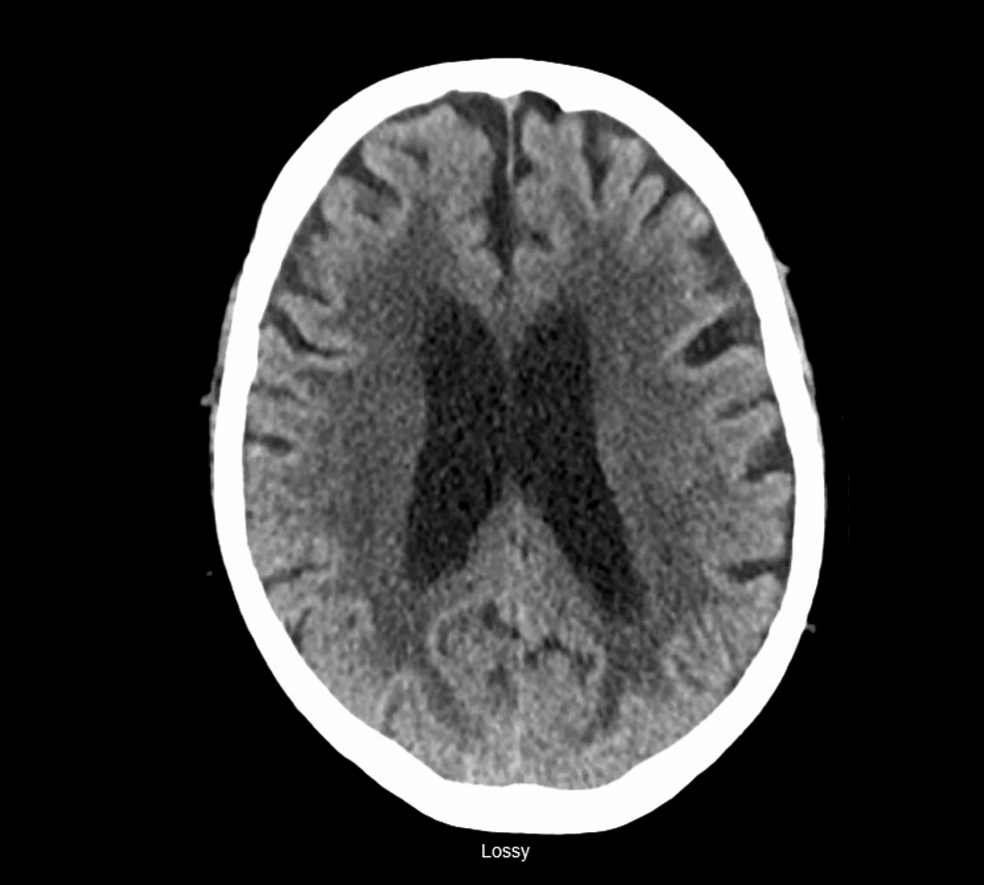
Normal CT scan of the head

**Figure 2:**
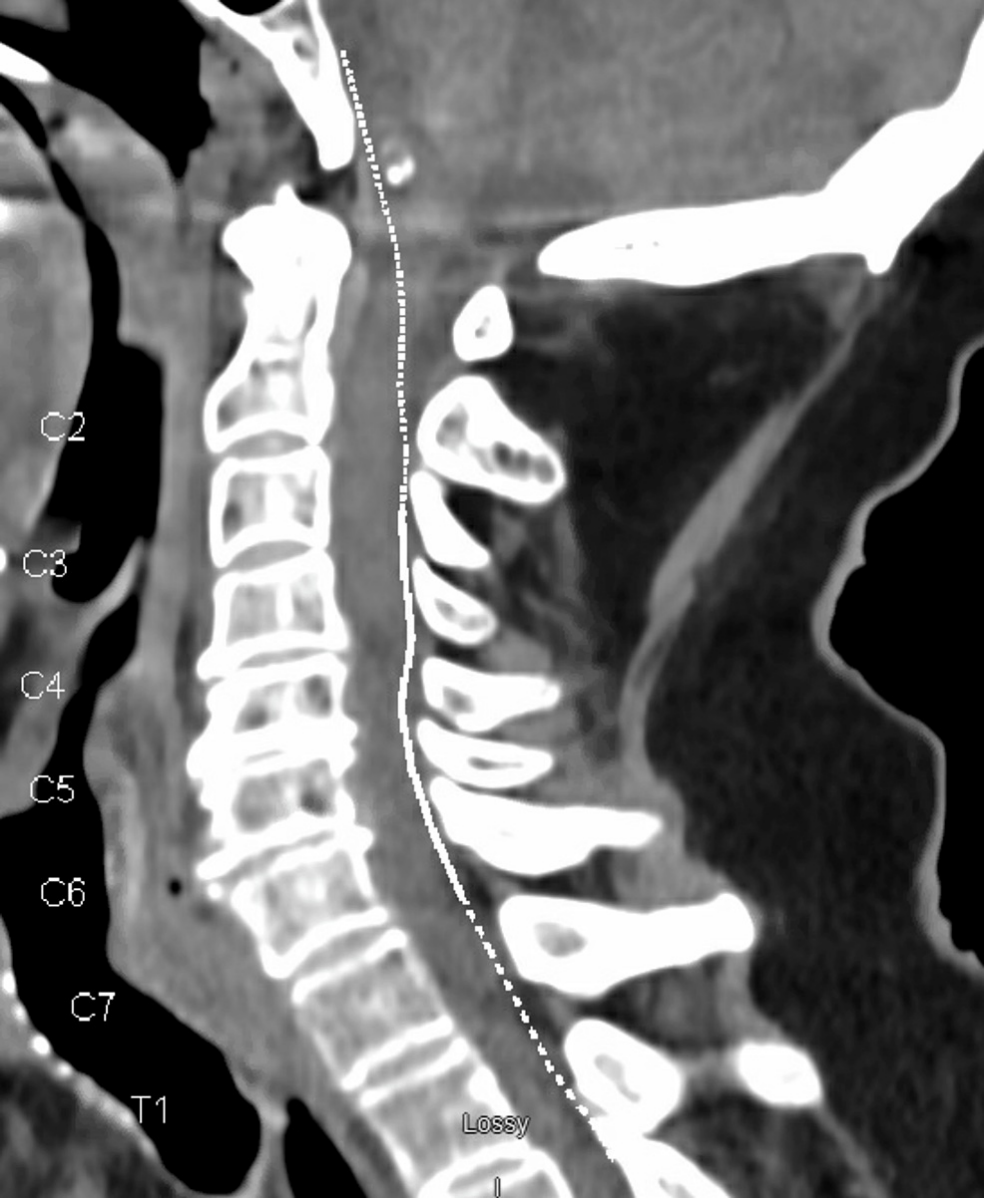
Normal CT scan of the cervical spine

**Figure 3:**
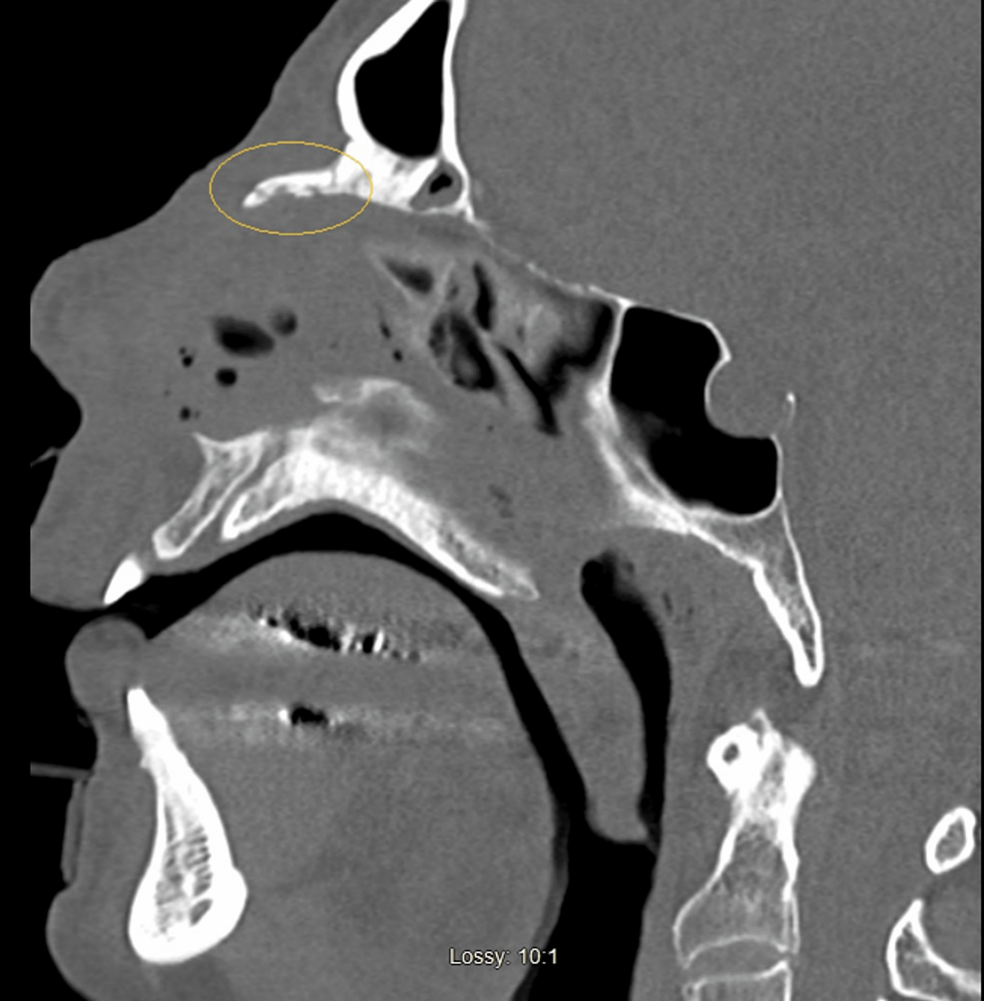
Nasal bone fracture

Similarly, urinalysis was negative for infection, and a computed tomography (CT) scan of the head and cervical spine was unremarkable for any acute intracranial bleeding and no cervical fractures (Figures 1-2), but a CT scan of the facial bones revealed subacute fractures in the nasal bones (Figure 3) for which plastic surgery was consulted and recommended a conservative approach without any surgical intervention.

During further workup for the reoccurrence of acute falls, thyroid-stimulating hormone (TSH) was found to be highly elevated at 29.8 uIU/mL (normal range: 0.47-4.68 uIU/mL) compared to previous levels of 1.37 uIU/mL two years ago; free thyroxine (T4) was found to be within low normal levels of 0.8 ng/dL (normal range: 0.8-2-2 ng/dL). Consequently, endocrinology was consulted and recommended levothyroxine 88 mcg and acquisition of thyroxine peroxidase antibody levels, which were negative to less than 9 IU/mL (normal range: 0-34 IU/mL). As a result, the patient was discharged to continued treatment, and oncology was made aware of the side effects caused by pembrolizumab.

## Discussion

The use of immune checkpoint inhibitors like pembrolizumab has become an integral component of the oncological therapeutic arsenal. However, given the recent implementation of these therapies, the exact mechanism of developing immune-related adverse events is not well-understood. Additionally, it is unclear if the development of a single immune-related adverse side effect predisposes patients to secondary undesired side effects during their clinical course. This case study illustrates the concurrent development of multiple immune-related adverse side effects treated with pembrolizumab. First, the patient developed a seronegative myasthenia gravis that was diagnosed with positive electromyography and was attributed to pembrolizumab usage. Then, during this admission and given the history of multiple falls, TSH was found highly elevated with inappropriately low normal free T4 and negative thyroid peroxidase (TPO) antibodies leading to the diagnosis of pembrolizumab-induced hypothyroidism. As a result, further investigation of pembrolizumab’s side effect profile, especially autoimmune-related adverse effects, and the frequency of concurrent side effect accruement is warranted, so clinicians should be aware of the untoward effects of these new biological drugs. Therefore, such findings will greatly impact clinical management and therapeutic decision-making for physicians and patients alike in the future.

## Conclusions

This case report presents a patient who was previously diagnosed with seronegative, pembrolizumab-induced myasthenia gravis and admitted after a mechanical fall. During further workup, it was evident that his TSH was highly elevated with a low normal T4 level, suggesting a diagnosis of hypothyroidism that was attributed to pembrolizumab usage.
